# The microbiological and clinical outcome of guide wire exchanged versus newly inserted antimicrobial surface treated central venous catheters

**DOI:** 10.1186/cc12867

**Published:** 2013-09-03

**Authors:** Nisha Parbat, Norelle Sherry, Rinaldo Bellomo, Antoine G Schneider, Neil J Glassford, Paul DR Johnson, Michael Bailey

**Affiliations:** 1 Department of Intensive Care, Austin Hospital, Heidelberg, Melbourne, VIC, Australia; 2 Departments of Microbiology and Infectious Diseases, Austin Hospital, Heidelberg, Melbourne, VIC, Australia; 3 ANZIC Research Centre, School of Public Health & Preventive Medicine, Monash University and Department of Medicine, The University of Melbourne, Melbourne, VIC, Australia

**Keywords:** central venous catheters, catheter related blood stream infection, sepsis, bacteraemia, intensive care, Gram negative bacteria

## Abstract

**Introduction:**

The management of suspected central venous catheter (CVC)-related sepsis by guide wire exchange (GWX) is not recommended. However, GWX for new antimicrobial surface treated (AST) triple lumen CVCs has never been studied. We aimed to compare the microbiological outcome of triple lumen AST CVCs inserted by GWX (GWX-CVCs) with newly inserted triple lumen AST CVCs (NI-CVCs).

**Methods:**

We studied a cohort of 145 consecutive patients with GWX-CVCs and contemporaneous site-matched control cohort of 163 patients with NI-CVCs in a tertiary intensive care unit (ICU).

**Results:**

GWX-CVC and NI-CVC patients were similar for mean age (58.7 vs. 62.2 years), gender (88 (60.7%) vs. 98 (60.5%) male) and illness severity on admission (mean Acute Physiology and Chronic Health Evaluation (APACHE) III: 71.3 vs. 72.2). However, GWX patients had longer median ICU lengths of stay (12.2 vs. 4.4 days; *P *< 0.001) and median hospital lengths of stay (30.7 vs. 18.0 days; *P *< 0.001). There was no significant difference with regard to the number of CVC tips with bacterial or fungal pathogen colonization among GWX-CVCs vs. NI-CVCs (5 (2.5%) vs. 6 (7.4%); *P *= 0.90). Catheter-associated blood stream infection (CA-BSI) occurred in 2 (1.4%) GWX patients compared with 3 (1.8%) NI-CVC patients (*P *= 0.75). There was no significant difference in hospital mortality (35 (24.1%) vs. 48 (29.4%); *P *= 0.29).

**Conclusions:**

GWX-CVCs and NI-CVCs had similar rates of tip colonization at removal, CA-BSI and mortality. If the CVC removed by GWX is colonized, a new CVC must then be inserted at another site. In selected ICU patients at higher central vein puncture risk receiving AST CVCs GWX may be an acceptable initial approach to line insertion.

## Introduction

Central venous catheters (CVCs) are commonly used in intensive care units. However, their use is not without risk [1-3] and line-related bacteraemia and sepsis remain significant problems [4-6]. Several studies in the 1990s suggested that regular line change does not decrease the risk of line-related sepsis and exposes patients to the risks associated with a new insertion (pneumothorax and arterial puncture) [7-9]. Thus, in the absence of clear identification of an alternative source of sepsis, clinicians currently respond to signs of possible or suspected CVC infection (new fever, increased white cell count, increased C-reactive protein or procalcitonin levels or a combination of these indicators) [10-12] by removing the CVC as the possible infective source [13,14]. However, if a CVC continues to be required, the removed CVC can then be replaced by two techniques: either by inserting a new CVC at another site or by inserting the new CVC at the same site by guide wire exchange (GWX) [9-15]. Finally, if GWX is performed and the tip of the line so removed grows a pathogen, the CVC inserted by GWX is removed and a fresh CVC is inserted at another site.

Theoretically, a new CVC insertion at a new site exposes the patient to some risk of arterial or lung puncture while, logically, GWX will eliminate the risk associated with such a puncture [16-19]. However, GWX may expose patients to a greater risk of line contamination through inadequate protection of the insertion field with anti-bacterial solutions. This, in turn, could potentially increase the risk of subsequent line tip colonization and bacteraemia [20-23]. Regrettably, despite the relatively common occurrence of suspected line related sepsis and the importance of this issue in daily practice, little empirical data exist to guide clinical practice.

The last decade has seen the widespread introduction of antimicrobial surface treated (AST) CVCs [24-27]. These AST-CVCs are associated with less line-related colonization and sepsis [26,27]. These CVCs may be logically expected to also diminish the risk of infection associated with GWX. Yet, to our knowledge, no studies have compared the risk of line colonization with pathogens and/or catheter-associated bloodstream infection (CA-BSI) when such surface treated CVCs are inserted by GWX. Accordingly, we conducted a retrospective study and estimated the incidence of subsequent line colonization and CA-BSI after GWX of AST-CVCs compared with a matched cohort of newly inserted AST-CVCs. We aimed to test the hypothesis that the incidence of CVC colonization with pathogens would still be much greater with GWX insertion.

## Materials and methods

This study was approved by the Austin Hospital Human Research Ethics Committee (HREC). The Austin HREC waived the need for informed consent for this study because it involved no intervention and the use of routinely collected data which were also de-identified and made anonymous for the purpose of the investigation.

Our unit has exclusively used antimicrobial surface treated (chlorhexidine acetate and silver sulfadiazine) triple lumen CVCs (Arrowgard blue^®^, Arrow-Howes, Reading, PA, USA) since 2005. Thus, we conducted a retrospective study of all CVCs inserted in our ICU as consecutively recorded in our database since 2005.

All line insertion-related data are recorded prospectively in our database as part of an ongoing quality assurance process whenever an ICU doctor inserts a CVC, arterial line, dialysis catheter or other invasive lines. The database contains specific information regarding when the CVC was inserted, the reason and method of insertion, any complications and the reason and timing of removal.

We used this database to identify all patients who had a central line inserted by GWX and selected a control population. Controls were selected as the patients in the database who had a new CVC inserted in ICU but not by the GWX method on the same day or as close to the same day as possible and, whenever possible, at the same site. This matching approach was selected because the average time to GWX of a CVC was seven days after admission and matching by admission to characteristics did not appear logical. On the other hand, matching controls for date of CVC insertion was considered to decrease the more relevant risks of CVC-insertion doctor and time-related bias.

We excluded patients who had a new CVC inserted (non-GWX) within 72 hours of a GWX, to avoid possible difficulties in attributing any source of bacteraemia. The decision to perform a GWX insertion in the presence of suspected CVC infection was made by the treating clinician in consideration of all possible risks and benefits of such an approach following unit protocol (see below).

### Approach to suspected line infection

During the study period, the approach to suspected line infection followed these unit guidelines:

1. Consider line infection if there is: a) a new fever or worsening fever not explained by likely infection elsewhere; b) an elevated white cell count or worsening white cell count not explained by likely infection elsewhere.

2. If line infection is suspected, inspect the insertion site. If there is redness or any suspicion of site infection, remove the line and insert a new CVC elsewhere. If there is no evidence of skin inflammation, consider the risks and benefits of GWX vs. new line insertion elsewhere.

3. If the patient has coagulopathy, thrombocytopenia, marked obesity, lack of other suitable sites for CVC insertion or other anatomical features that make new line insertion relatively contraindicated, proceed to GXW.

4. In all cases, send the line of the suspected tip for culture and obtain blood cultures, urine cultures and sputum cultures.

5. If GWX has been completed and the line tip of the removed CVC is subsequently found to be colonized, remove the GWX CVC and insert a new CVC elsewhere.

6. If a CVC is removed because it is no longer needed and there is no clinical suspicion of infection, do not send the tip for culture.

Patients with shock (vasopressor dependence) were excluded as they required continued infusion of vasopressors, which could not be maintained during the GWX of the line.

We then obtained data on CVC tip cultures for both the GWX and control groups using the hospital microbiology database, as well as blood cultures taken during the time the line was *in situ *and for 48 hours after line removal.

Once the intervention and control groups had been identified, we compared the two groups for patient characteristics as obtained by the ICU admissions and discharges database, which is part of the Australian and New Zealand adult patient database program [28]. Such data included age, sex, admission diagnosis, Acute Physiology and Chronic Health Evaluation (APACHE) III score, use of mechanical ventilation and key outcomes. Similarly, we compared the two groups for microbiological outcomes including: a) the number of CVC tips that were sent for culture, b) the number which had a positive tip culture for a pathogen (exclusion of skin flora and skin commensal organisms unless isolated from two different blood cultures as per Centre for Disease Control guidelines), and c) the number of patients who had catheter-associated blood stream infection (CA-BSI) using the criteria described by the US National Nosocomial Infections Surveillance System of the Centers for Disease Control and Prevention as used in a recent multicentre study [29]. Finally, we compared patient outcomes. For this comparison, we assessed the patients' length of stay in the ICU, the length of stay in hospital, and their survival status at ICU and hospital discharge.

### Technique of guide wire exchange (GWX)

**Figure 1:**
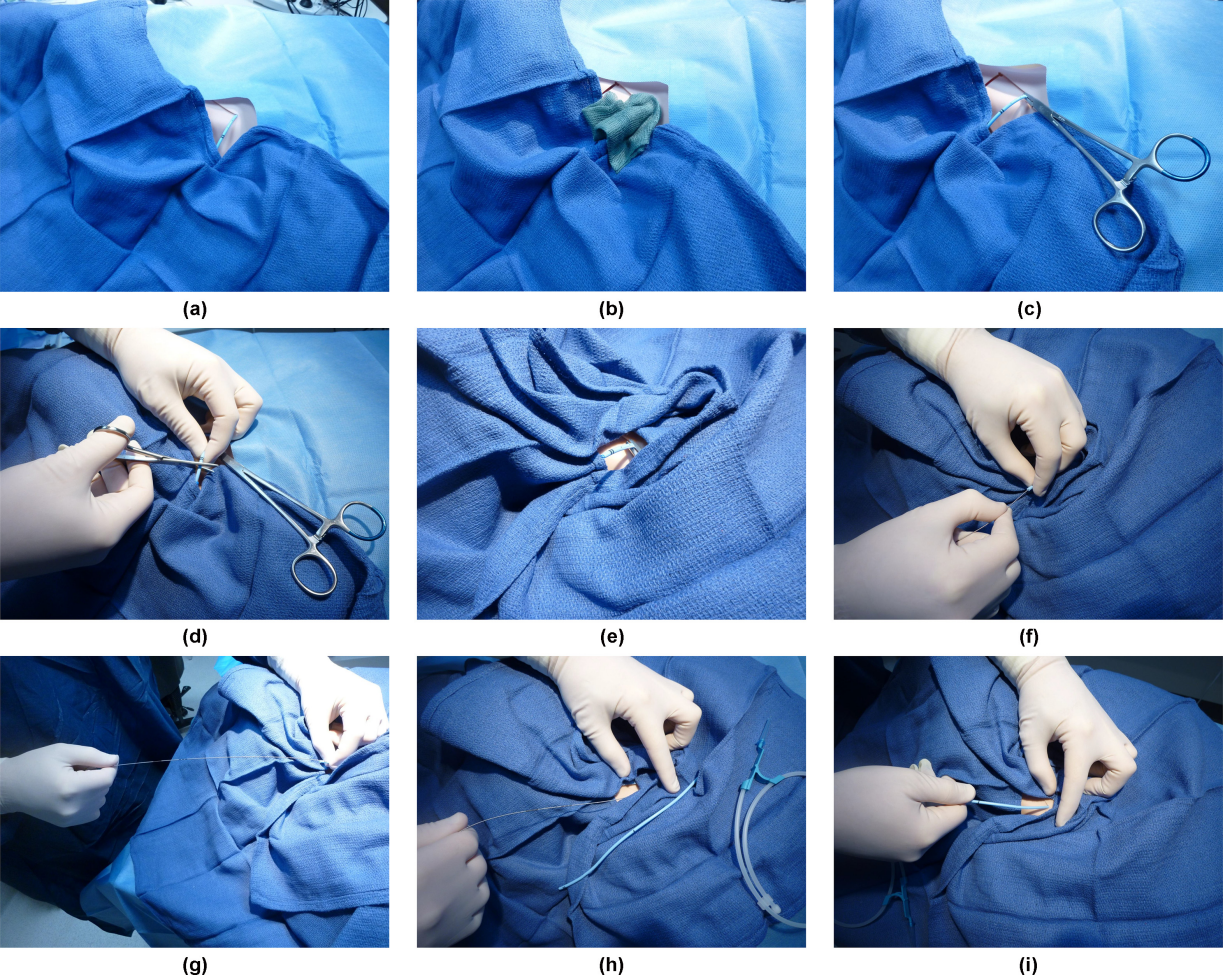
**Illustration of the sequence for the guide wire exchange of central venous catheter**. (**a**) Approximately 5 cm of the catheter external to the entry site are extensively prepared with chlorhexidine solution in 70% alcohol solution. Some parts of the catheter are more difficult to cover with the antiseptic solution. These, including the hub and connecting ports, are covered with drapes and later removed. (**b**) The skin surrounding the catheter insertion site is also treated with an alcohol and chlorhexidine solution, and sterile drapes are placed appropriately to cover the catheter except for the component under manipulation. The segment of catheter remaining uncovered is then covered with alcohol-chlorhexidine impregnated gauze. (**c**) The catheter is clamped just above its point of entry in the skin and, using sterile scissors. (**d**) The CVC is cut below the hub and 3 to 4 cm from the skin. (**e**) The external cut portion is covered with drapes. The operator then removes his/her gloves and changes to a new pair of sterile gloves. The field is further covered so that only a small skin surface of 3 to 5 cm^2 ^is exposed where the CVC enters the skin. (**f**) The severed cross section of the line shows the various lumens and the soft J tip of the guide wire is then inserted into the largest lumen (distal 16 gauge lumen) until it meets the resistance of the clamp. The clamp is removed with one hand, while the other holds the external part of the CVC. The wire is then advanced to 20 cm and the 'old' CVC is removed over it. (**g**) The operator now has a wire in place for a new central line insertion. (**h**) The removed CVC is placed on to the sterile drapes, the distal 5 cm portion is cut with sterile scissors, placed in a sterile container and set for subsequent microbiological culture. (**i**) A new catheter can now be inserted over the wire and secured to the skin as would be the case for a new CVC.

Since 1995, the technique employed at our institution for changing CVCs over a guide wire involves full aseptic precautions (gloves, gown, hat and mask). The steps used to insert the line by GWX are shown in a photographic sequence using a mannequin from Figure 1a-i.

In brief, the patient is positioned as for a new CVC insertion. Approximately 5 cm of the catheter external to the entry site are extensively prepared with chlorhexidine solution in 70% alcohol solution. Some parts of the catheter are more difficult to cover with the antiseptic solution. These, including the hub and connecting ports, are covered with drapes, which are later removed.

The skin surrounding the catheter insertion site is also treated with an alcohol and chlorhexidine solution, and sterile drapes are placed appropriately to cover the catheter except for the component under manipulation. The segment of catheter remaining uncovered is then covered with alcohol-chlorhexidine impregnated gauze. The catheter is clamped just above its point of entry in the skin and, using sterile scissors, the CVC is then cut below the hub and 3 to 4 cm from the skin. The external cut portion is covered with drapes. The operator then removes his/her gloves and changes to a new pair of sterile gloves.

The field is further covered so that only a small skin surface of 3 to 5 cm^2 ^is exposed where the CVC enters the skin. The severed cross-section of the line shows the various lumens and the soft J tip of the guide wire is then inserted into the largest lumen (distal 16 gauge lumen) until it meets the resistance of the clamp. The clamp is removed with one hand, while the other holds the external part of the CVC.

The wire is then advanced to 20 cm and the 'old' CVC is removed over it. The removed CVC is placed onto the sterile drapes, the distal 5 cm portion is cut with sterile scissors, placed in a sterile container and sent for subsequent microbiological culture. The operator now has a wire in place for a new central line insertion. A new catheter can now be inserted over the wire and secured to the skin as would be the case for a new CVC.

All lines are inserted by a variable group of doctors (residents, fellows, specialists) during the study period. If junior doctors inserted the catheter, it is under supervision of a senior doctor.

**Figure 2:**
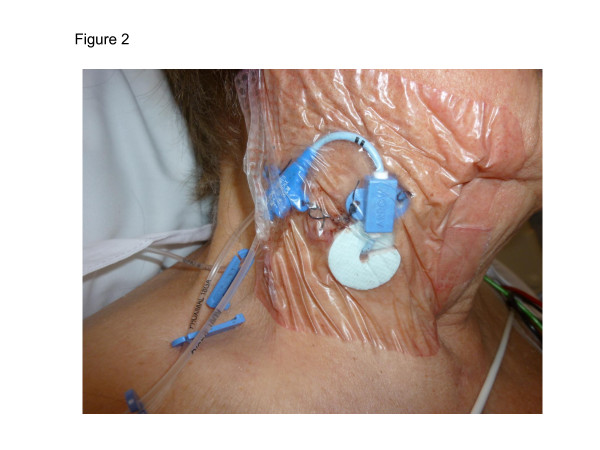
**Illustration of central venous catheter insertion site care and cover Skin preparation was with 2% chlorhexidine in 70% alcohol**. Protection of the CVC site after insertion was in the form of a "sandwich" cover with a transparent dressing (IV 300 Frame Delivery, Smith & Nephew, Hull, UK) and with a chlorhexidine impregnated antimicrobial disk (Biopatch, Bridgewater, Ethicon Inc., NJ, USA) around the skin entry site.

Skin preparation is with 2% chlorhexidine in 70% alcohol. Protection of the CVC site after insertion is in the form of a "sandwich" cover with a transparent dressing (IV 300 Frame Delivery, Smith & Nephew, Hull, UK) and with a chlorhexidine impregnated antimicrobial disk (Biopatch, Ethicon Inc., Bridgewater, NJ, USA) around the skin entry site (Figure 2). The dressing is not changed until line removal; nursing care is given on a 1:1 ratio to all patients with no regular change to giving sets.

Line removal is decided upon by the treating clinicians with consideration of all relevant aspects of patient management.

### Microbiological methods

In our microbiology lab, CVC tips are processed by first adding 2 ml of D/E Neutralizing Broth (Neogen, Lansing, MI, USA) to neutralize any residual disinfectants or antimicrobials. The tip and broth are vortexed for 30 seconds, then 100 μL of the fluid are inoculated onto a horse blood agar plate (BioMerieux, Marcy-l'Etoile, France). Plates are incubated at 36°C in a 5% CO_2 _incubator, and read at 24 and 48 hours. Any positive cultures are subsequently identified by routine laboratory methods, and reported as qualitative cultures only.

Blood samples are collected in aerobic and anaerobic bottles for culture (Bactec 9240, Becton Dickinson, Sparks, MD, USA), and cultured for five days. Positive bottles are then sub-cultured manually onto HBA, chocolate HBA and anaerobic HBA (plus Sabaraud dextrose agar if yeasts present in Gram stain) (BioMerieux), and incubated aerobically, anaerobically and in 5% CO_2 _for up to five days. Positive cultures are subsequently identified according to routine laboratory methods.

### Statistical analysis

All analyses are performed using SAS version 9.2 (SAS Institute Inc., Cary, NC, USA). Group comparisons are made using chi-square tests for equal proportions of Fisher's exact test when appropriate for categorical data, Student's *t*-test and Wilcoxon rank sum test for continuous data. Results are reported as numbers (percentages), means (standard deviation) or medians (interquartile range) as appropriate. Due to the low number of outcomes, multivariable logistic regression was not performed. A two-sided *P*-value of 0.05 was considered to be statistically significant.

## Results

We compared microbiological data from the 145 patients with a GWX-CVC insertion with those of 163 control patients with a NI-CVC from December 2005 to February 2011 (Table 1). During this time a total of 2,378 CVCs were inserted in the unit. At the time of CVC insertion, all patients receiving GWX have suspected line infection. All lines replaced by GWX were sent for cultures and blood cultures were obtained. The line tips of the CVC removed by GWX grew coagulase negative staphylococci in 11 cases, enterococci in 1, gram negative bacilli in 1 and *Candida albicans *in 1. In all cases, the newly inserted GWX line was removed and a new CVC placed elsewhere. In two additional cases, both blood culture and CVC tip grew the same organisms (*Klebsiella *and *Candida albicans*). In both cases, the newly inserted GWX CVC was removed and a new CVC placed elsewhere.

**Table 1:** Study patients' characteristics at ICU admission

		Total (*n *= 308)	Controls (*n *= 163)	GWX (*n *= 145)	*P *
**Sex**	Male	186 (60%)	98 (60%)	88 (61%)	0.92
**Age**	Mean (SD)	60.6 (16.4)	62.21 (15.6)	58.7 (17.1)	0.07
**Diagnosis at admission**	Respiratory	40 (13%)	24 (15%)	16 (11%)	0.33
	Hepatic	25 (8.1%)	12 (7%)	13 (9%)	0.60
	Cardiac	77 (25%)	44 (14.3%)	33 (23%)	0.39
	GI	43 (14%)	23 (27%)	20 (14%)	0.93
	Hematological	5 (1.6%)	1 (0.6%)	4 (3%)	0.19
	Neurological	33 (10.7%)	23 (27%)	10 (7%)	0.04
	Metabolic	6 (2%)	4 (2.5%)	2 (1%)	0.68
	Sepsis	40 (13%)	29 (18%)	11 (8%)	0.01
	Renal	12 (4%)	4 (2.5%)	8 (6%)	0.23
	Vascular	10 (3.25%)	5 (3%)	5 (3%)	0.85
	Trauma	16 (5.2%)	4 (2.5%)	12 (8%)	0.36
	Musculoskeletal	1 (0.3%)	1 (0.6%)	0 (0%)	0.99
**Apache III scores**	Mean (SD)	71.8 (13.6)	72.3 (30.44)	71.3 (30.8)	0.77
**Co-morbidities**					
	Immune disease	31 (10%)	20 (12%)	12 (8%)	0.26
	Immuno-suppression	28 (9%)	12 (7%)	15 (10%)	0.35
	Chronic liver disease	25 (8%)	13 (8%)	11 (8%)	0.91
	Metastases	12 (4%)	9 (6%)	4 (3%)	0.23
	Leukaemia/myeloma/lymphoma	9 (3%)	6 (4%)	4 (3%)	0.14
	Hepatic failure	12 (4%)	6 (4%)	5 (3%)	0.92
	IDDM	0 (0%)	6 (4%)	2 (1%)	0.21
	Chronic respiratory disease	3 (1%)	0 (0%)	2 (1%)	0.22
	Cardiovascular disease	12 (4%)	4 (2%)	8 (6%)	0.16
	Chronic renal failure	18 (6%)	12 (7%)	6 (4%)	0.23

GWX, guide wire exchange; IDDM, Insulin-Dependent Diabetes Mellitus

Both groups were similar for gender distribution, mean age and APACHE III scores on admission. There was also no significant difference in APACHE III admission-related diagnostic groups and co-morbidities. However, 134 (92.4%) patients in the GWX group were mechanically ventilated compared with 127 (77.9%) in the control group (*P *= 0.005). There were no complications of GWX insertion. In the control group, there were two episodes of arterial puncture which required manual compression with one residual haematoma. There were no pneumothoraces. Microbiological data are presented in Table 2.

**Table 2:** Microbiological outcomes

	Controls	GWX group	*P*-value
**No. of patients who had blood cultures taken**	121 (74%)	106 (73%)	0.83
**No. of positive peripheral blood culture results **	31 (26%)	26 (25%)	0.85
**No. of line tips cultured**	95 (58%)	85 (56%)	0.78
**No. of cultured line tips growing pathogens among patients where CVC tip was sent for culture**	6 (6.3%)	5 (5.9%)	0.90
**No. of patients with same organism in blood culture and line tip**	3 (1.8%)	2 (1.4%)	0.77

CVC, central venous catheter

As expected given a period of pre-GWX cannulation, there was a significant difference in ICU length of stay between the two study groups. The GWX group had a longer ICU length of stay than the control group (median (IQR) of 12.2 (4.8 to 17.8) days compared to 4.4 (2.1 to 9.0) days in the control group; *P *< 0.001. This significant difference persisted for hospital length of stay with a median for the GWX group of 30.7 (17.3 to 56.7) days compared with 18.0 (8.5 to 37.7) days for the control group (*P *< 0.001). Median time from insertion to discharge from ICU was 5 (2 to 10) vs. 3 (2 to 7) days for the GWX and control group, respectively (*P *= 0.005); while time from insertion to hospital discharge was 19 (10 to 42) vs. 13 days (5 to 31) (*P *= 0.003).

The most common site for line insertion was the internal jugular vein in both groups (91 (62.7%) in the GWX group, vs. 96 (58.9%) in the control group; *P *= 0.49) followed by the femoral vein (25 (17.2%) in the GWX group vs. 42 (25.7%) in the control group; *P *= 0.07) and finally, the subclavian vein (8 (5.5%) in the GWX group vs. 9 (5.5%) in the control group; *P *= 0.99]

Tip cultures from the CVC were obtained in 81 (55.9%) GWX patients and in 95 (58.3%) of controls (*P *= 0.66). Pathogens were isolated from six (6.3%; 95% CI: 1.7 to 10.9) tips (three Gram negative organisms, two *Staphylococcus *isolates and one *Candida*) in six control patients compared with five (5.9%; 95% CI: 1.9 to 9.9%) tips in five GWX patients (five Gram negative isolates) (Table 2).

For the 13 patients who had a positive CVC tip culture and a positive blood culture during their ICU stay, the same organism was isolated in both cultures in two patients in the GWX group vs. three patients in the control group (Table 2). The overall incidence of CA-BSI was 2.6 events/1,000 catheter days (1.8 (0 to 5) for GWX vs. 3.5 (0 to 14) for controls. There was no significant difference between the two groups for ICU mortality (19 (13.1%) vs. 31 (19.0%); *P *= 0.15) and hospital mortality (35 (24.1%) vs. 48 (29.4%); *P *= 0.29).

## Discussion

### Key findings

In a clinical environment where all CVCs inserted are antimicrobial surface treated (AST) CVCs, we conducted a retrospective observational study to test the hypothesis that GWX would result in a major increased incidence of subsequent line colonization with pathogens compared with NI-CVCs. Our results suggest that, in selected ICU patients at higher central vein puncture risk, using AST-CVCs and the GWX method carries similar risks of subsequent line colonization with pathogens and CA-BSI as NI-CVCs. Survival to both hospital and ICU discharge were also similar for both groups.

### Previous studies

GWX might increase the risk of catheter-related infections [30-32]. However, previous studies did not adjust for all relevant confounders or report how GWX was performed. Moreover, current guidelines on GWX [33] are based on data from studies published in the 1990s. In the most prominent of these studies [7], the GWX procedure was often performed with insertion via the most distal port and distal to the CVC hub (Sawyer R and Peled H, personal communication). In 2000, on the basis of such information, a review concluded that GWX might be associated with a higher rate of catheter colonisation and catheter-related bacteraemia [13]. In addition, recent guidelines [33] imply that GWX would be the final step in the therapeutic process for a suspected CVC-related infection.

In contrast to the above positions, several other observational studies reported that GWX of a CVC was microbiologically safe [11,15,30,34]. Unfortunately, in the absence of randomized trials or contemporaneous controls, these reports were confounded by selection bias and provided only weak evidence for or against GWX-CVCs. In the last 10 years, only two studies have assessed the potential relationship between GWX and microbiological outcome. One [15] reported a rate of colonization for newly inserted CVCs of 6.8% compared with 2.3% for GWX CVCs. No information was provided on the technique of GWX. The other found that GWX was associated with an increased risk of catheter-related bacteraemia [35]. However, this latter study only assessed 76 CVCs inserted by GWX, with only half being triple lumen CVCs. Moreover, one-third was inserted outside the ICU, chlorhexidine preparation of the site was only used in less than 10% of insertions, and AST-CVCs were not used. Additionally, in contrast to our study, the process of GWX insertion was not described.

### Implications of study findings

The results of our study suggest that, when using AST-CVCs and a sterile procedure for insertion of the guide wire below the hub and directly into the AST catheter, CVC replacement by GWX in the ICU is likely microbiologically equivalent to *de novo *CVC insertion. The information obtained in our study justifies the use of GWX as an acceptable approach to CVC replacement in selected ICU patients deemed at high risk from attempted central vein puncture because of coagulopathy, thrombocytopenia and/or anatomical characteristics (for example, extreme obesity, poor central vein visualization on ultrasound), provided that AST CVCs are used and a sterile technique with insertion of the wire below the hub is applied and there is no evidence of insertion site inflammation. It must be emphatically stated, however, that, if the line exchanged by the guide wire is found to be colonized, the GWX CVC must then be removed and a new CVC inserted at another site.

### Strengths and limitations

This study has several strengths. It describes the largest cohort of GWX CVCs in the literature. It is the first to assess the microbiological outcome of GWX of AST-CVCs and to explicitly present the technique of GWX in detail. Nonetheless, our database provided only information on the admission diagnosis but not on the presence, degree and alternative sources of sepsis at the time of CVC removal. These shortcomings applied to both cohorts similarly, making the introduction of bias in favour or against GWX unlikely.

Our study compared microbiological outcomes and major clinical outcomes with those of contemporaneous newly inserted CVCs. However, the comparison with new CVCs inserted early in the course of the ICU admissions in a cohort of patients with a relatively short stay biased the study against GWX-CVCs. The fact that, despite such negative bias, there was no evidence of greater colonization rates after GWX supports the notion that GXW insertion of AST-CVCs is likely microbiologically similar to newly inserted CVCs.

Our study data were obtained from the ICU and microbiology databases. These were collected prospectively and electronically recorded and were not amenable to manipulation or *post-hoc *ascertainment bias. The study period was long, allowing the capture of data from many different clinicians (junior and senior), which suggests a degree of generalizability. However, our study was retrospective with all the inherent limitations associated with such design. Of technical importance, we applied a specific technique to GWX as illustrated in the figures. This technique may have decreased the risk of colonization. Unfortunately, all previous papers describing the microbiological outcome of GWX failed to report the details of the GWX technique.

Our findings are from an intensive care unit, which serves as a referral centre for liver transplantation and which, therefore, handles many immune-suppressed patients with advanced liver disease and coagulopathy, who require CVCs for extended periods and are at high risk of bleeding. Thus, our results may not apply to different patient populations. We acknowledge that CVC insertion in higher risk patients may still be safe under ultrasound guidance. However, logically the avoidance of puncture and dilatation must provide a greater degree of safety.

The rates of CA-BSI appear slightly higher than those reported in a recent multicentre study [29]. However, the study population had twice our unit average stay among controls and six times the unit average among GWX patients. Our ICU applied all components of the bundle of line insertion care [29] during the entire study period in a strategic attempt to decrease noscomial infection associated with any cannulation in complex patients [36,37].

Our findings could represent a type II error. However, ours is the largest cohort of patients receiving GWX of CVC where all catheters in question were triple lumen AST-CVCs catheters. Finally, the point estimate for line colonization with pathogens and CA-BSI was lower in the GWX cohort despite the illness duration related bias against them. Thus, within these statistical limitations, our study provides initial evidence for clinicians to estimate the microbiological risk of GWX insertion of CVCs in selected patients at higher risk of central vein cannulation.

## Conclusions

In conclusion, GWX of an antimicrobial surface treated CVC in selected patients at high risk from central vein cannulation appears not to be associated with an increased rate of bacteraemia or pathogen colonization compared with a newly inserted CVC. Such GWX of a CVC is safe and easy to perform. Treatment of suspected CVC-related sepsis should include antibiotics and removal of the CVC. However, if the CVC needs replacement, in selected patients with severe coagulopathy or unfavourable anatomy or limited availability of other insertion sites, clinicians may now consider GWX using an AST-CVC with a clearer appreciation of its microbiological risk.

## Key messages

• In selected patients, central venous catheter replacement by guide wire exchange is microbiologically associated with a low rate of line-related bacteraemia when using antimicrobial surface-treated catheters.

• In selected patients, guide wire exchange of central venous catheters can be technically performed in a systematic way that minimizes risk of colonization.

• In selected patients at high risk of bleeding or with anatomically unfavourable characteristics, replacement of a new central venous catheter by guide-wire exchange may be a safe and preferred approach when antimicrobial surface treated catheters are used.

• If the CVC removed by GWX is found to have been colonized, however, the CVC inserted by GWX must be removed and a new CVC must be inserted at another site.

## Abbreviations

APACHE: Acute Physiology and Chronic Health Evaluation; AST: antimicrobial surface treated; CA-BSI: catheter associated bloodstream infection; CVC: central venous catheter; GWX: guide wire exchange; GWX-CVC: CVCs inserted by GWX; HREC: Human Research Ethics Committee; NI-CVC: newly inserted triple lumen AST CVCs.

## Competing interests

The authors declare that they have no competing interests.

## Authors' contributions

All authors made substantial contributions to the conception and design of the study, or to the acquisition of data or analysis and interpretation of data. NP was in charge of the clinical data collection and wrote the first draft of the manuscript. NS was in charge of the microbiological data collection. RB was in charge of the design of the study, assisted with analysis and revised and critiqued the manuscript until ready. AGS was in charge of assisting with the clinical data collection and manuscript development. NJG assisted with the clinical outcome data collection and manuscript development. PDRJ assisted with the microbiological data collection and manuscript development and revision from a microbiological point of view. MB was in charge of the statistical analysis and study design development from a statistical point of view and reviewed the manuscript for content and accuracy. All authors have given final approval of the manuscript version to be published.
